# Assessing the structure of the posterior visual pathway in bilateral macular degeneration

**DOI:** 10.1038/s41598-023-31819-x

**Published:** 2023-03-27

**Authors:** Holly D. H. Brown, Richard P. Gale, André D. Gouws, Richard J. W. Vernon, Archana Airody, Rachel L. W. Hanson, Heidi A. Baseler, Antony B. Morland

**Affiliations:** 1 Centre for Cognition and Neuroscience, Department of Psychology, University of Huddersfield, Huddersfield, UK; 2 Department of Psychology, University of York, York, UK; 3 York Neuroimaging Centre, University of York, York, UK; 4 York Biomedical Research Institute, University of York, York, UK; 5 Hull York Medical School, University of York, York, UK; 6 Academic Unit of Ophthalmology, York and Scarborough Teaching Hospital NHS Foundation Trust, York, UK

**Keywords:** Visual system, Macular degeneration, Macular degeneration

## Abstract

Macular degeneration (MD) embodies a collection of disorders causing a progressive loss of central vision. Cross-sectional MRI studies have revealed structural changes in the grey and white matter in the posterior visual pathway in MD but there remains a need to understand how such changes progress over time. To that end we assessed the posterior pathway, characterising the visual cortex and optic radiations over a ~ 2-year period in MD patients and controls. We performed cross-sectional and longitudinal analysis of the former. Reduced cortical thickness and white matter integrity were observed in patients compared to controls, replicating previous findings. While faster, neither the rate of thinning in visual cortex nor the reduction in white matter integrity during the ~ 2-year period reached significance. We also measured cortical myelin density; cross-sectional data showed this was higher in patients than controls, likely as a result of greater thinning of non-myelinated tissue in patients. However, we also found evidence of a greater rate of loss of myelin density in the occipital pole in the patient group indicating that the posterior visual pathway is at risk in established MD. Taken together, our results revealed a broad decline in grey and white matter in the posterior visual pathway in bilateral MD; cortical thickness and fractional anisotropy show hints of an accelerated rate of loss also, with larger effects emerging in the occipital pole.

## Introduction

Macular degeneration results in the loss of function of light sensitive photoreceptors and a consequent loss of vision. Recently, techniques that aim to restore light sensitivity have been investigated and appear promising. However, during the period of time in which vision was lost, the integrity of the posterior visual system, the optic radiations and visual cortex, may also change. We have found cross sectional evidence of atrophic changes in the cortex, while others have reported a loss in integrity of the optic radiations. In this study, we have acquired data over a 2-year period to determine whether there are changes to the visual cortex and optic radiations over time.

In cases of vision loss, atrophy of the posterior visual pathway, greater than that observed with ageing has been reported^1–13^. Given the retinotopic organisation of visual cortex, when macular degeneration (MD) is present in both eyes, resulting in overlapping visual defects, the cortical representation of the visual defect lacks sensory input; reductions in cortical thickness and volume in this representation have been reported in both juvenile MD (JMD) and age-related MD (AMD)^5,11,12,14–17^.

In addition to assessments of grey matter thickness and volume, advances in neuroimaging have allowed for the evaluation of more subtle changes in microstructure, macrostructure and anatomical connectivity in vivo, making it a valuable technique to assess cortical myelin and white matter changes in detail^10,18–22^. A combination of diffusion properties can help clarify the state of white matter tracts in both health and disease, providing more information than volumetric measures alone^23^. In the case of central vision loss specifically, previous research has shown a reduction in white matter integrity in the optic radiations^10,18,22^. As yet, longitudinal assessments of the integrity of the optic radiations in bilateral MD have not been conducted.

New methods using an in vivo T1- and T2-weighted MRI ratio have been developed to derive estimates of cortical myelin^24^. Neurodegenerative conditions such as multiple sclerosis—where observed cognitive and physical decline is a result of demyelination—highlight the fundamental role of myelin in the function of the central nervous system and how the grey and white matter can both be impacted by disease progression^25,26^. Animal models have shown that rats deprived of visual input to one eye exhibit a decrease in cortical myelination in the deprived region of visual cortex^27^. While changes in cortical thickness and volume have largely been observed in MD in humans, changes in myelination have not yet been explored, but could help to better characterise the progression of MD and the nature of corresponding changes in visual cortex.

The current study aimed to provide a thorough assessment of the state of the posterior visual pathway in patients with bilateral macular degeneration (age-related and juvenile forms), compared to sighted controls. We are the first to our knowledge to present both cross-sectional and longitudinal assessments of cortical measures (thickness and myelin density) as well as white matter integrity, specifically reporting fractional anisotropy (FA), radial diffusivity (RD) and axial diffusivity (AD) all in the same cohort of patients and controls. We aimed to determine whether both grey and white matter changes occur in patients with long-standing MD over a 2-year period, or whether brain structure remains stable. Our rationale for seeing changes in our 2-year period was driven previous work published by the lab^17^, cortical changes were observed in unilateral AMD patients after a ~ 5-year period, but not in the short term (approximately 3–4 months). What remains to be investigated is how the posterior visual pathway changes in the later stages of the disease. Taken together, a 2-year follow up period should be informative on the atrophic changes to the posterior visual system resulting from visual loss. As cortical myelin density is yet to be explored in MD, we asked whether previously reported changes in the optic radiations may coincide with changes in the cortex in the form of reduced myelin density. It was predicted that the larger cortical and white matter abnormalities would be observed in the regions of interest (ROIs) that capture central visual field representations (occipital pole), but in the absence of assessments of patient scotomas, we could not rule out the possibility that more subtle differences from normal may be observed in representations of more peripheral visual field locations (calcarine sulcus).

## Methods

### Participants

Twelve individuals with macular degeneration (MD) were recruited from either the Ophthalmology department at York Teaching Hospital or through advertisements in sight loss support groups in York (referred to as MD1–MD12; 6 males and 6 females, mean age = 71.42 years, age range = 28–87 years). MD patients all had a diagnosis of bilateral macular degeneration at the time of the study and thus all have a confirmed loss of input from the central retina to the brain. During the screening process, patients were excluded if they had any other eye-affecting or neurological pathologies. The aetiology of their visual loss is heterogeneous; two individuals have juvenile MD, and 9 individuals have AMD (dry, wet or both—see Table 1 for details). This was an opportunity sample and as such, further details regarding the time since disease onset at the time of enrolment as well as formal clinical measures characterising the scotoma and severity of the disease are limited because the National Health Service requires that costs outside routine treatment are met, which was not possible as part of this PhD work. However, we were able to obtain estimates of the time since disease onset and visual acuity scores either from the individual (for those with JMD and dry AMD) or the eye clinic at York Teaching Hospital (for those with AMD who attended this clinic for treatment). It is important to note, however, that even in a clinical sample of patients with similar disease characteristics, the start of disease can only ever be approximate as pre-symptomatic features may exist long before clinical presentation. Therefore, the aim was to measure brain changes and rates of change across a range of patients with established central visual loss. Patients were scanned between one and six times over a ~ 2-year period (see Table 1 for patient details). MD12 was included in the cross-sectional analysis, but excluded from longitudinal analyses due to ill health preventing participation in follow up MRI sessions.

**Table 1:** MD participant demographics. Some missing data for *time since disease onset* and *visual acuity*—participants were unable to provide this information, or we were unable to access it.

Group	Eye disease	Receiving treatment	Age at enrolment (years)	Time since disease onset (years)	Visual acuity ETDRS letters [logMAR] OD	Visual acuity ETDRS letters [logMAR] OS	Number of MRI sessions	Time between first and last MRI session (years)
MD1	Wet AMD	Yes	85	–	40 [0.90]	76 [0.18]	3	0.57
MD2	Wet AMD	Yes	86	–	39 [0.92]	60 [0.50]	3	0.47
MD3	Wet AMD	Yes	76	3.6	69 [0.32]	54 [0.62]	5	1.68
MD4	Dry AMD	No	79	5	34 [1.02]	34 [1.02]	6	2.08
MD5	Wet AMD	Yes	87	–	55 [0.60]	79 [0.12]	3	0.47
MD6	JMD (Best's Disease)	No	51	20	85 [0.00]	85 [0.00]	6	2.07
MD7	Wet and Dry AMD	Yes	73	0.5	67 [0.36]	0 [2.20]	5	1.56
MD8	Dry AMD	No	78	14.9	0 [2.20]	44 [0.82]	4	1.02
MD9	Dry AMD	No	68	–	40 [0.90]	44 [0.82]	2	0.66
MD10	Dry AMD	No	65	21.2	65 [0.40]	65 [0.40]	4	1.61
MD11	JMD (Stargardt Disease)	No	28	1	90 [− 0.10]	90 [− 0.10]	4	0.98
MD12	Wet and Dry AMD	No	81	23.4	–	–	1	0
Average			71.42	11.20			3.83	1.10

Eighteen sighted controls (referred to as C1–C18, 8 males and 10 females, mean age = 55.33 years, age range = 24–73 years. For the subset of participants C1-C12, mean age = 49.83 years, age range = 24–73 years) were recruited for this study through advertisements at the York Neuroimaging Centre (YNiC), University of York. One control participant was excluded from the DTI analysis due to difficulty identifying optic radiations bilaterally.

Finally, an additional 15 sighted controls (referred to as C19-C33, 10 males and 5 females, mean age = 66.27 years, age range = 48–83 years) were recruited through advertisements at YNiC. Despite being scanned with a different MRI protocol, they were included in cross-sectional analysis to assess the relationship between our two measures: cortical thickness and myelin density.

All participants (patients and controls) were screened to determine if they were eligible for an MRI scan. Participants were excluded if they had a pacemaker or pacing wires, cochlear implant, spinal or neural stimulator, programmable hydrocephalus shunt, or shrapnel in their body. Any medical implants (such as hip or knee replacements) were checked by the MRI operator to determine if they were safe. Finally, all participants were also asked if they were able to lie comfortably in a supine position for up to 45 min.

Written informed consent was obtained from all participants. This study followed the tenets of the Declaration of Helsinki with approval granted by YNiC Research, Ethics and Governance Committee and the NHS Research Ethics Committee (IRAS: 158456).

### MRI data acquisition

For MD1–MD12 and C1–C18, scanning was performed at the University of York Neuroimaging Centre using a 3 Tesla HD MRI system (GE Signa Excite 3.0 T, High resolution brain array, MRI Devices Corp., Gainesville, FL) with an 8 channel whole head High Resolution Brain Array.

#### MD1–MD12 and C1–C12

For each time point for each participant, one 8-channel 3D T1-weighted anatomical image (TR = 7.88 ms, TE = 2.99 ms, TI = 600 ms, voxel size = 1 × 1 × 1 mm^3^, flip angle = 10°, matrix size 256 × 256 × 176, FOV = 256 mm) and one T2-weighted anatomical image was acquired (TR = 2500 ms, TE = 75.48 ms, voxel size = 1 × 1 × 1 mm^3^, flip angle = 90°, matrix size = 256 × 256 × 176, FOV = 256 mm). All T1- and T2-weighted images were gradwarped (a system-specific correction) to correct for nonlinearities in the gradients.

Two diffusion weighted MRI scans were acquired in each session, with opposing phase encoding directions. These were identical, with just the phase encoding direction changing (P/A to A/P). The duration of the individual dMRI scans was ~ 6 min. A single-shot pulsed gradient spin-echo echo-planar imaging sequence was used with the following parameters: *b* = 1000 s/mm^2^, 21 unique diffusion directions, 47 slices, FOV = 192 mm, TR = 12 s, TE = 88.5 ms (minimum full), voxel size = 2 × 2 × 2 mm^3^, matrix = 96 × 96, flip angle = 90°. Three volumes without diffusion weighting (b0) were acquired at the start of each scan.

#### C13–C18

For each time point for a subset of controls (n = 6) one 8-channel 3D FSPGR T1-weighted anatomical image was acquired (TR = 7.76 ms, TE = 2.96 ms, TI = 450 ms, voxel size = 1.13 × 1.13 × 1 mm^3^, flip angle = 20°, matrix size = 256 × 256 × 176, FOV = 290 mm). All T1-weighted images were gradwarped (a system-specific correction) to correct for nonlinearities in the gradients.

Two diffusion scans were acquired in each session. The phase-encoding reversal scan was shorter (~ 2 min versus ~ 11 min) as it was used to correct for distortion only, hence only 6 diffusion sampling directions were acquired for this scan. Broadly, protocols were: b = 1000 s/mm^2^, 45 directions, 58 slices, FOV = 192 mm, TR = 12 s, TE = 87.1 ms (minimum full), voxel size = 2 × 2 × 2 mm^3^, matrix size = 96 × 96, flip angle = 90°. 3 volumes without diffusion (b0) were acquired at the start of the diffusion sequence. As we were replicating protocols from the first time point which were conducted by different research groups for this subgroup of participants, it was essential to keep protocols consistent within individuals to reduce the effect of scan type on the outcome measures. Due to an error in the first time point for the phase-encoding reversal scan whereby up-sampling was not turned off, two participants have slightly different parameters (voxel size 0.75 × 0.75 × 0.75 mm^3^, matrix size = 256 × 256).

#### C19–C33

Scanning was performed on the 3 T Magnetom Prisma MR scanner (Siemens Healthineers, Erlangen, Germany), using the 20-channel head/neck receive-array coil. Participants were scanned using the Human Connectome Project (HCP) recommended protocols^28^. One T1-weighted anatomical image was acquired using a 3D-MPRAGE sequence (TR = 2400 ms, TE = 2.28 ms, TI = 1010 ms, voxel size = 0.8 × 0.8 × 0.8 mm^3^, flip angle = 8°, matrix size = 320 × 320 × 208, FOV = 256 mm) and one T2-weighted anatomical image was acquired (TR = 3200 ms, TE = 563 ms, voxel size = 0.8 × 0.8 × 0.8 mm^3^, flip angle = 120°, matrix size = 320 × 320 × 208, FOV = 256 mm). No diffusion data were acquired for this group of controls.

### Longitudinal experiment

#### Study design (MD1–MD12, C1–C18)

MD participants were enrolled in the study for up to ~ 2-year with a maximum of 6 MRI scans during this period. MD patients were given the option to opt in for the sessions they wished to attend, hence not all participants completed all sessions offered. All 18 controls were scanned twice, between 1.67 and 2.42 years apart (mean time = 2.2 years). See Table 1 for details of the number of sessions completed by each MD participant.

#### Region of interest selection

**Figure 1:**
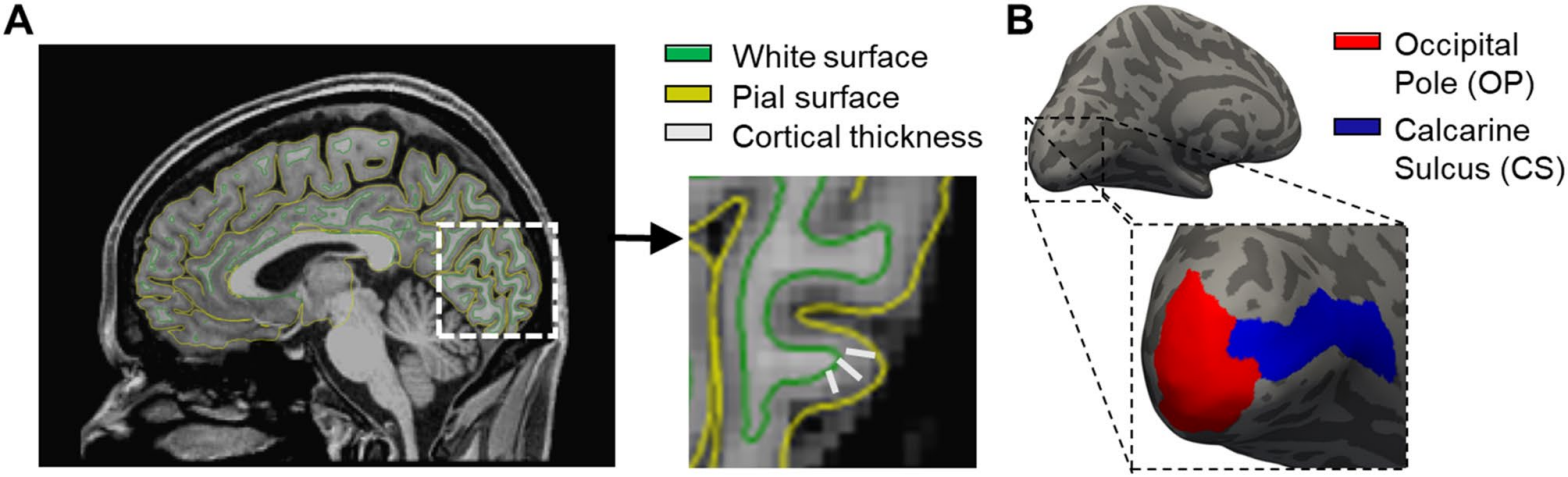
(**A**) Processed anatomical data in the sagittal view, zooming in on occipital regions. Cortical thickness (grey lines) is calculated by taking the shortest distance (mm) between the white matter surface (green) and the grey matter/pial surface (yellow). (**B**) Our regions of interest (ROIs) shown on the inflated cortical surface: Occipital pole (red) and calcarine sulcus (blue) taken from FreeSurfer’s Destrieux atlas.

ROIs were defined using FreeSurfer’s Destrieux anatomical atlas^29^. We used the occipital pole (parcellation index = 42) and the calcarine sulcus (parcellation index = 44) consistent with those used by Hanson et al.^16,17^. These ROIs were used for cortical thickness and myelin density analyses and are shown on the inflated surface in Fig. 1B. For the diffusion MRI analysis, ROIs were first converted from fsaverage6 surface labels into a volume, before being transformed from the fsaverage space into MNI152 1 mm space using FSL’s FLIRT^30^. ROIs were used as waypoints to guide the tractography analysis from LGN to early visual cortex.

#### Cortical thickness processing

All T1- and T2-weighted data (where possible) were analysed using the automatic longitudinal processing stream^31^ in FreeSurfer analysis suite (version 6, available at: http://surfer.nmr.mgh.harvard.edu/). To compute reliable estimates of the rate of change in cortical thickness, several processing steps were completed. Each individual data set was processed automatically using FreeSurfer; Cortical reconstruction and volumetric segmentation were performed, and segmentations were inspected and manually corrected where applicable. Once individual time points were checked and processed, an unbiased within-subjects template space and image was created^32^ using robust inverse consistent registration^33^. Next, skull stripping, Talairach transforms, atlas registration as well as spherical surface maps and parcellations were initialised using common information for the within-subject template created. This is known to significantly increase the reliability of cortical thickness measures and in turn increase the statistical power^31^. Cortical thickness was calculated by taking the shortest distance (millimetres) between the white matter surface (where it meets the grey matter) and the grey matter (pial) surface at each vertex across the cortex; measurements across all vertices within an ROI were then averaged (Fig. 1A).

#### Myelin density processing

To compute cortical myelin density, T1- and T2-weighted data were processed using the automatic pipeline developed by the Human Connectome Project (HCP, version 4.0.0) which uses the T1w/T2w ratio to generate myelin maps^28^. The HCP MRI data pre-processing pipelines use tools from FreeSurfer^34^ and FSL^35^. The pipeline generates cortical myelin maps in the subject’s native space^36^. As there is currently no comparable longitudinal processing stream for generating myelin maps, myelin density data was analysed statistically to separate within- and between subjects effects. However, as this was a FreeSurfer based analysis, the same ROIs could be computed for this separate data analysis. MD1–M11 and C1–C12 were included in this analysis. No T2-weighted images were acquired for C13–C18, and therefore estimates of myelin density could not be computed for this group.

#### Cross-sectional analysis—thickness and myelin

To visualise the impact of group on both myelin density and cortical thickness, as well as the relationship between measures, we calculated group mean values for MD patients (MD1–MD11) and controls (C1–C18 for cortical thickness and C1–C12 for myelin density) for both ROIs. We simply averaged across hemispheres and timepoints for each individual, before then computing a group average.

For C19–C33, data were processed using the HCP minimal processing stream described above, after which the cortical thickness and myelin values were extracted from the subject’s native space. The ROIs were computed as described previously. Myelin density and cortical thickness were converted to Z-scores for this due to the fact data were acquired on different MRI scanners, and given the difference in resolution, thickness and myelin values will differ. C19–C33 were only included in the correlation analysis to assess the relationship between cortical thickness and myelin density.

#### Rate of change calculation—thickness and myelin

Rate of change in cortical thickness across all time points was calculated and plotted for both ROIs. Values were averaged across hemispheres. Rate of change was calculated for myelin density using the slope of a regression line based on the times measurements were taken (x-values), and the individual myelin values at each time point (y-values). This was done for every individual before the group result was calculated.

#### Probabilistic tractography

Probabilistic fiber tracking allows for automatic extraction of the white matter tracts of interest in both hemispheres, and we did this using the FSL (v6.0) toolbox Xtract^37^. Before running Xtract, we fit the crossing fibers model^38^ to the corrected diffusion data using FDT’s bedpostx (Bayesian Estimation of Diffusion Parameters Obtained using Sampling Techniques). This calculates the number of crossing fibers within each voxel. All default parameters were used when running this model except for drawing 10,000 streamlines (or samples) in each voxel (default is 5000). We then registered our FA data from the subject's native diffusion space to a standard 1 mm^3^ space using FNIRT nonlinear registration^39^. The target image used was the FMRIB58_FA which is in the same space as the MNI152 standard space image. The registration steps that are part of the full Tract-Based Spatial Statistics (TBSS)^40^, part of FSL^41^, have been optimised, and so we used these to avoid doing manual registrations. Xtract also requires the inverse warp that would allow data to go from MNI standard space back to the native diffusion space, so to generate this we used FSL’s invwarp command on the transformation generated in the prior step.

**Figure 2:**
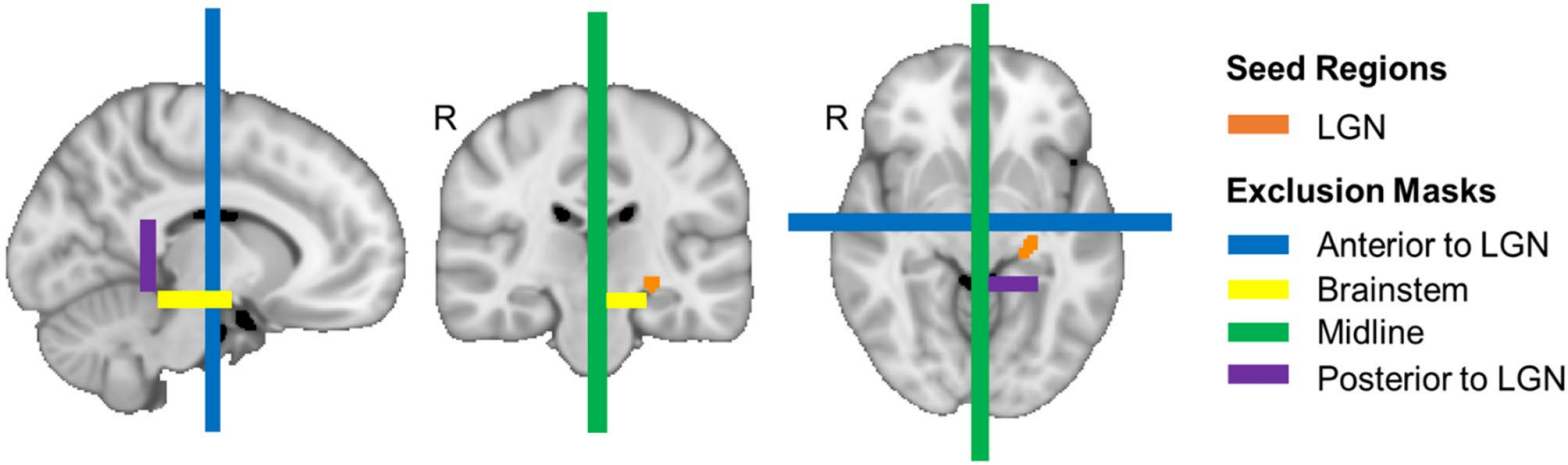
Example exclusion points and seed regions for the left optic radiations overlaid on the MNI152_1mm standard brain^37^. The lateral geniculate nucleus (LGN) was our seed region in each hemisphere (left shown in orange). Our exclusion masks included: a region anterior to the LGN to avoid longitudinal fibers being included (blue), the brainstem (yellow), the midline (green) and a region posterior to the LGN (purple) to help capture the curvature of the optic radiations close to the LGN. Exclusion points were the mirrored along the midline for the right hemisphere for the LGN, posterior point (purple) and brainstem points (yellow).

Probabilistic tractography is run on the bedpostx output; the aim of probtrackx2^42,43^ is to produce streamlines that originate in a pre-defined seed region—the LGN for this study—that also fulfil criteria such as passing through a waypoint (our ROIs in visual cortex). Exclusion points are also included to filter out any irrelevant projections—see Fig. 2 for details.

#### Fiber tract cleaning

Before extracting our diffusion measures, we cleaned up the tracts to help reduce noise induced by complex fiber orientations. First, we set a conservative threshold to exclude voxels where fewer than 20% of fibers passed through; each tract was visually inspected for spurious branching and at a threshold of 20% further manual pruning of tracts was not necessary. Doing this gives us greater confidence that fibers are indeed part of a genuine white matter tract and meaningful metrics have been obtained^37,44^. We normalised our data and divided it into equally spaced bins (0–100) and extracted summary statistics from the central 80% of the tract; 10% of locations at the start (closer to the LGN) and end (early visual cortex) of the tracts were discarded to remove any noise and avoid partial-voluming effects. These locations have fibers going in many directions and the increased angle and/or curvature of the fibers adds to the noise. Similar methods have also been employed by previous work^8,22,45^. Tracts were identified in both hemispheres of 12 MD patients and 17 control participants. Fractional anisotropy (FA), axial diffusivity (AD) and radial diffusivity (RD) were plotted for multiple locations along each tract. Data were averaged across hemispheres.

#### Linear mixed effects modelling

To assess the effects of age, group, time and any group × time interactions for our three measures (cortical thickness, myelin density and FA), data for each measure at every time point were analysed using R statistical software version 3.5.2^46^ and the ‘lmerTest’ package^47^ to perform a linear mixed effects analysis^48,49^. We also wanted to determine if during the period of the study, there were any changes in FA over time in portions of the optic radiations that emerged significantly reduced in MD patients. The model (*R syntax*) used for each ROI or tract for each cortical thickness or FA was:

$$Measure \, \sim \, 1 \, + \, Age \, + \, Group:Time \, + \, \left( {1 | \, Participant} \right)$$

For myelin density, we added in cortical thickness as a fixed effect to determine whether this was a significant predictor of myelin density. The model used was:

$$Measure \, \sim \, 1 \, + \, Age \, + \, Group:Time \, + \, Thickness \, + \, \left( {1 | \, Participant} \right)$$

Age at the time of recruitment, thickness (for myelin density only), group and time were included as fixed effects, and participants as a random effect. By having this as a random effect, all participants have a different intercept, and this allows us to account for between-subject variability in cortical thickness, myelin density or fractional anisotropy. We examined both measures for both ROIs for both the MD and control groups. The model for each ROI was evaluated using restricted maximum likelihood (REML) estimation to ensure the model was robust. The significance was assessed using *t* tests which incorporated Satterthwaite's method to calculate our degrees of freedom and in turn control for possible type-1 errors^47^. Finally, the ‘effectsize’ package^50^ was used to calculate estimated effect sizes using R version 4.2.0^51^. In the ideal world we would have run the models with ROI as a factor to make inferences about differences of effects dependent on ROI. However, we found that a linear mixed effect model was not supported by the number of samples and therefore would not offer an appropriate solution. We therefore provide context to effects detected in the different ROIs by referring to effect sizes.

## Results

### Assessing the relationship between cortical thickness and myelin density in sighted controls

**Figure 3:**
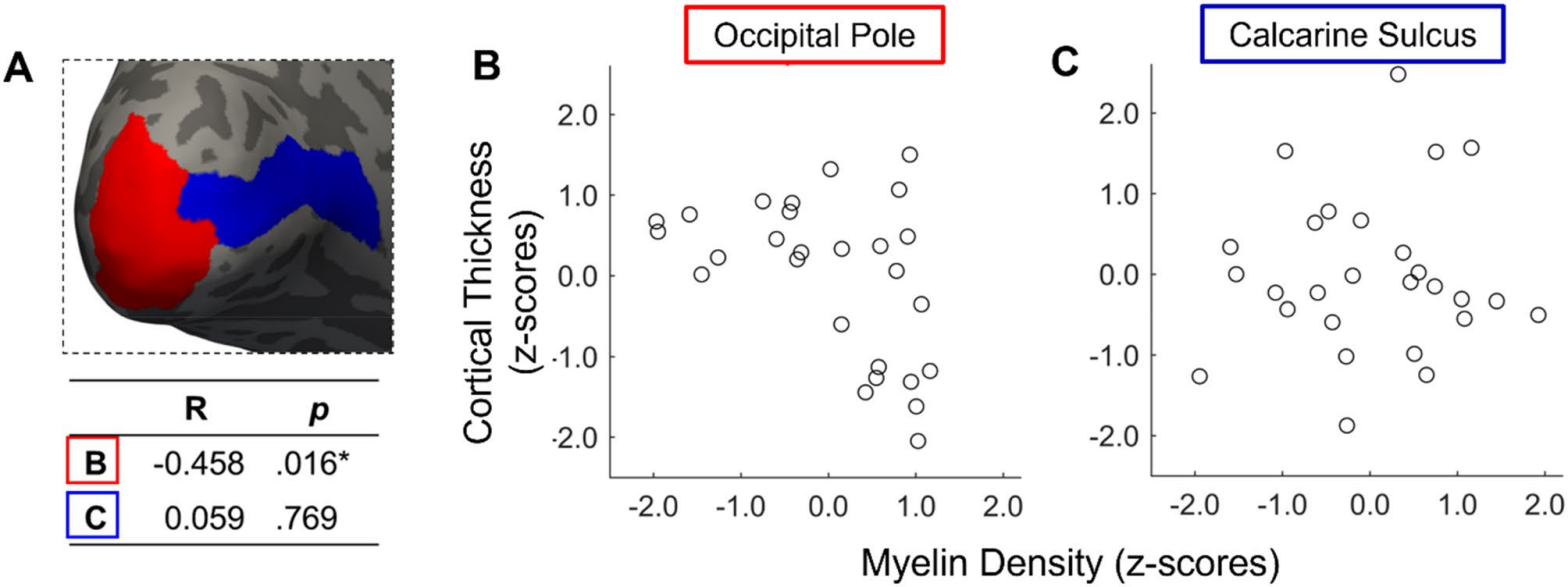
(**A**) Occipital pole (red) and Calcarine sulcus (blue) displayed on the inflated cortical surface with a summary of the correlation (R) and corresponding p values for each region. (**B**,**C**) Scatterplots displaying myelin density against cortical thickness (both converted to z-scores) for the occipital pole and calcarine sulcus respectively. All sighted controls who had both measures (C1–C12 and C19–C33; note that C13–C18 did not have a T2-weighted scan and therefore myelin density could not be calculated).

To understand the possible impact of macular degeneration (MD) on both of our measures, we pooled together all sighted control data to enable us to try and understand the nature of the relationship between the measures in normal ageing and provide us with the necessary context for any measured changes in myelin density. As described by Natu et al.^52^, it is possible that a higher myelin density value can emerge as a result of a reduction in cortical thickness, which drives the ratio artificially higher. However, if myelin density decreases with no correlated change in cortical thickness, then myelin density changes are likely legitimate. Given data were acquired using different MRI scanners, we converted cortical thickness and myelin density values to z-scores separately before combining them, to allow us to interrogate the data on the same scale (see Fig. 3). It appears there is a hint of a negative correlation between cortical thickness and myelin density in the occipital pole (*r* = − 0.458, *p* = 0.016), but not in the calcarine sulcus (*r* = 0.059, *p* = 0.769). Given this relationship we might encounter artificially high values of cortical myelin in those with MD, who have been previously shown to have thinner cortex.

#### Cortical thickness

**Figure 4:**
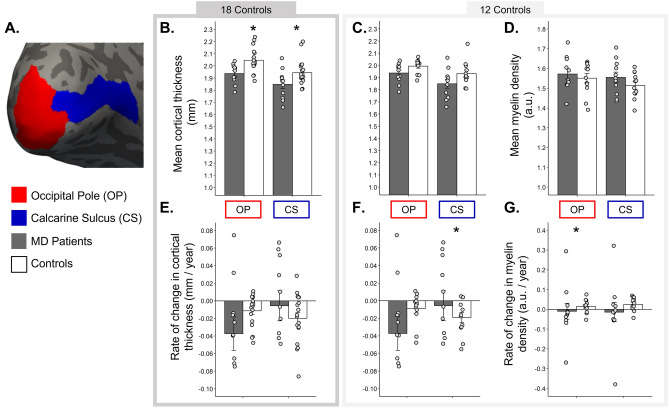
(**A**) Regions of interest (ROIs) on the inflated cortical surface for MD patient (black bars) and sighted controls (white bars). Error bars = SEM. (**B**,**C**) Mean cortical thickness (mm) for occipital pole and calcarine sulcus. (**B**) = MD patients and C1–C18. (**C**) = MD patients and C1–C12, the subset of controls that contribute myelin data. (**D**) Mean myelin density for MD patients and C1–C12. (**E**,**F**) Rate of change in cortical thickness (mm/year) for MD patients and C1–C18 (**E**) and MD patients and C1–C12 (**F**). (**G**) Rate of change in myelin density (units per year) for MD patients and C1-C12.

To establish whether results for the group of MD patients (MD1–M11) and sighted controls were consistent with previous cross-sectional studies, we compared the mean of all cortical thickness values obtained over time for each group for the occipital pole and calcarine sulcus (Fig. 4B,C). Table 2 condenses the results for the linear mixed effects models. Given the difference in the average age of our two groups, we wanted to account for the role it may play in any observed findings by including it as a fixed effect in our model. A significant effect of age was observed, showing that irrespective of group, age had a significant effect on cortical thickness in the occipital pole (*t*(25.76) = 2.09, *p* = 0.047, *d* = 0.82), but not the calcarine sulcus (*t*(25.96) = − 0.76, *p* = 0.453 *d* = -0.30). Despite this, we still find a significant effect of group, suggesting any group differences are likely attributed to visual impairment, not age. The effect of group was shown for both our ROIs (occipital pole: *t*(27.76) = − 3.52, *p* < 0.001, *d* = − 1.34, calcarine sulcus: *t*(26.92) = − 2.06 *p* = 0.050, *d* = − 0.79), indicating MD patients had, on average, a thinner cortex than sighted controls, and looking at the effect sizes this seems to be greater in the occipital pole—capturing the representation of the central visual field.

**Table 2:** Model output from the Linear Mixed Effects Models assessing changes in cortical thickness, myelin density and fractional anisotropy between MD patients and controls over time. Here we are reporting the fixed effects with the standard error (SE) for the predictors we used for each ROI (occipital pole and calcarine sulcus). Age refers to the age of participants at the first time point. Group refers to MD patients and sighted controls. Time refers to the time in years since the baseline scan was conducted. Thickness in the Myelin Density section refers to cortical thickness. *T* test results determine the significance of each of the predictors reported here. Estimates for effect size (Cohen’s D) and corresponding 95% confidence intervals are reported. All significant results are in bold.

Measure	Predictor	Occipital pole						Calcarine sulcus					
		b (SE)	t (df)	*p*	Effect size (Cohen's d)	95% Confidence intervals		b (SE)	t (df)	*p*	Effect size (Cohen's d)	95% Confidence intervals	
						Lower	Upper					Lower	Upper
Cortical thickness													
	Age	**0.002 (0.001)**	**2.09 (25.76)**	**0.047**	**0.82**	**0.01**	**1.62**	− 0.001 (0.001)	− 0.76 (25.96)	0.453	− 0.30	− 1.07	0.48
	Group	− **0.140 (0.04)**	− **3.52 (27.76)**	**0.00**	− **1.34**	− **2.15**	− **0.50**	− **0.099 (0.05)**	− **2.06 (26.92)**	**0.050**	− **0.79**	− **1.57**	**0.00**
	Time	− **0.012 (0.01)**	− **2.34 (50.00)**	**0.023**	− **0.66**	− **1.23**	− **0.09**	− **0.020 (0.005)**	− **4.30 (50.07)**	**0.000**	− **1.22**	− **1.81**	− **0.61**
	Group:Time	− 0.016 (0.01)	− 1.56 (50.54)	0.126	− 0.44	− 0.99	0.12	0.016 (0.01)	1.64 (50.32)	0.107	0.46	− 0.10	1.02
Myelin density													
	Age	0.001 (0.001)	0.97 (21.47)	0.342	0.42	− 0.44	1.27	− 0.002 (0.001)	− 1.58 (19.56)	0.130	− 0.71	− 1.62	0.21
	Thickness	− 0.201 (0.19)	− 1.04 (54.95)	0.301	− 0.28	− 0.81	0.25	− 0.04 (0.15)	− 0.24 (25.87)	0.810	− 0.09	− 0.86	0.68
	Group	0.021 (0.05)	0.46 (28.76)	0.651	0.17	− 0.56	0.90	**0.11 (0.04)**	**2.52 (29.30)**	**0.017**	**0.93**	**0.16**	**1.69**
	Time	0.014 (0.01)	1.28 (46.41)	0.207	0.38	− 0.21	0.95	0.03 (0.01)	1.99 (48.95)	0.052	0.57	− 0.01	1.14
	Group:Time	− **0.047 (0.02)**	− **2.32 (47.55)**	**0.025**	− **0.67**	− **1.25**	− **0.09**	− 0.04 (0.02)	− 1.88 (48.16)	0.066	− 0.54	− 1.11	0.04
Fractional anisotropy													
	Age	− **0.002 (0.0004)**	− **6.55 (24.99)**	**0.000**	− **2.62**	− **3.67**	− **1.54**	− **0.002 (0.0004)**	− **6.24 (25.13)**	**0.000**	− **2.49**	− **3.51**	− **1.43**
	Group	− **0.030 (0.0138)**	− **2.21 (26.15)**	**0.036**	− **0.86**	− **1.66**	− **0.05**	− **0.029 (0.0137)**	− **2.11 (26.38)**	**0.044**	− **0.82**	− **1.61**	− **0.02**
	Time	− 0.002 (0.0015)	− 1.26 (49.12)	0.214	− 0.36	− 0.92	0.21	− 0.002 (0.0015)	− 1.04 (49.26)	0.302	− 0.30	− 0.86	0.27
	Group:Time	− 0.004 (0.0031)	− 1.37 (49.43)	0.178	− 0.39	− 0.95	0.18	− 0.001 (0.0031)	− 0.54 (49.59)	0.590	− 0.15	− 0.71	0.40

Next, we evaluated changes in cortical thickness over time. As part of the FreeSurfer longitudinal pipeline, rate of change in cortical thickness was calculated for both ROIs, allowing us to visualise how cortical thickness values may change over the ~ 2-year period in both groups. Figure 4 shows the rate of change in cortical thickness for the occipital pole and calcarine sulcus (Fig. 4E,F).

A significant effect of time emerged in the linear mixed effects model highlighting that irrespective of group, with time, cortical thickness declines (occipital pole: *t*(50.00) = − 2.34, *p* = 0.023, *d* = − 0.66 and calcarine sulcus: *t*(50.07) = − 4.30, *p* < 0.001, *d* = − 1.22). While MD patients appear to show a faster decline in cortical thickness within the occipital pole (Fig. 4D,E), we did not observe a significant group × time interaction (occipital pole: *t*(50.54) = − 1.56, *p* = 0.126, *d* = − 0.44 and calcarine sulcus: *t*(50.32) = 1.64, *p* = 0.107, *d* = − 0.46).

#### Myelin density

Table 2 condenses the results for the linear mixed effects models. As with cortical thickness, age was entered as a predictor into our model, however this was not a significant predictor of myelin density for either ROI (occipital pole: *t*(21.47) = 0.97, *p* = 0.342, *d* = 0.42 and calcarine sulcus: *t*(19.56) = − 1.58, *p* = 0.130, *d* = − 0.71). While we observed a greater mean myelin density value for our MD patients compared to our sighted control group in both ROIs (Fig. 4D), the linear mixed effects model revealed a significant effect of group for the calcarine sulcus only (occipital pole: *t*(28.76) = 0.46, *p* = 0.651, *d* = 0.30 and calcarine sulcus: *t*(29.30) = 2.52, *p* = 0.017, *d* = 0.93), with myelin density higher for MD patients.

We also assessed changes in myelin density over time. The rate of change in myelin density for each ROI is shown in Fig. 4G. Data appear to show a decline in myelin density over time in MD patients across both ROIs, but in contrast, a small increase was observed for the control group. This result for the controls appears to complement the cross-sectional findings between the two measures and the increase observed could simply be explained by an artefact caused by the decrease in cortical thickness. For MD patients, this is not the case. Time was not a significant predictor of myelin density for either ROI. Looking at our rate of change plots (Fig. 4G), this is likely driven by the control data. In terms of group × time interactions, a significant effect emerged for the occipital pole (*t*(47.55) = − 2.32, *p* = 0.025, *d* = − 0.67; calcarine sulcus: *t*(48.16) = − 1.88, *p* = 0.066, *d* = − 0.54). The estimate for this predictor being negative (− 0.04) indicates that MD patients decline faster than controls for the occipital pole. Finally, to assess whether cortical thickness measures were a predictor of myelin density, we entered thickness into our model; this was not significant for either of our ROIs.

### Analysis of white matter tracts

#### Fractional anisotropy

**Figure 5:**
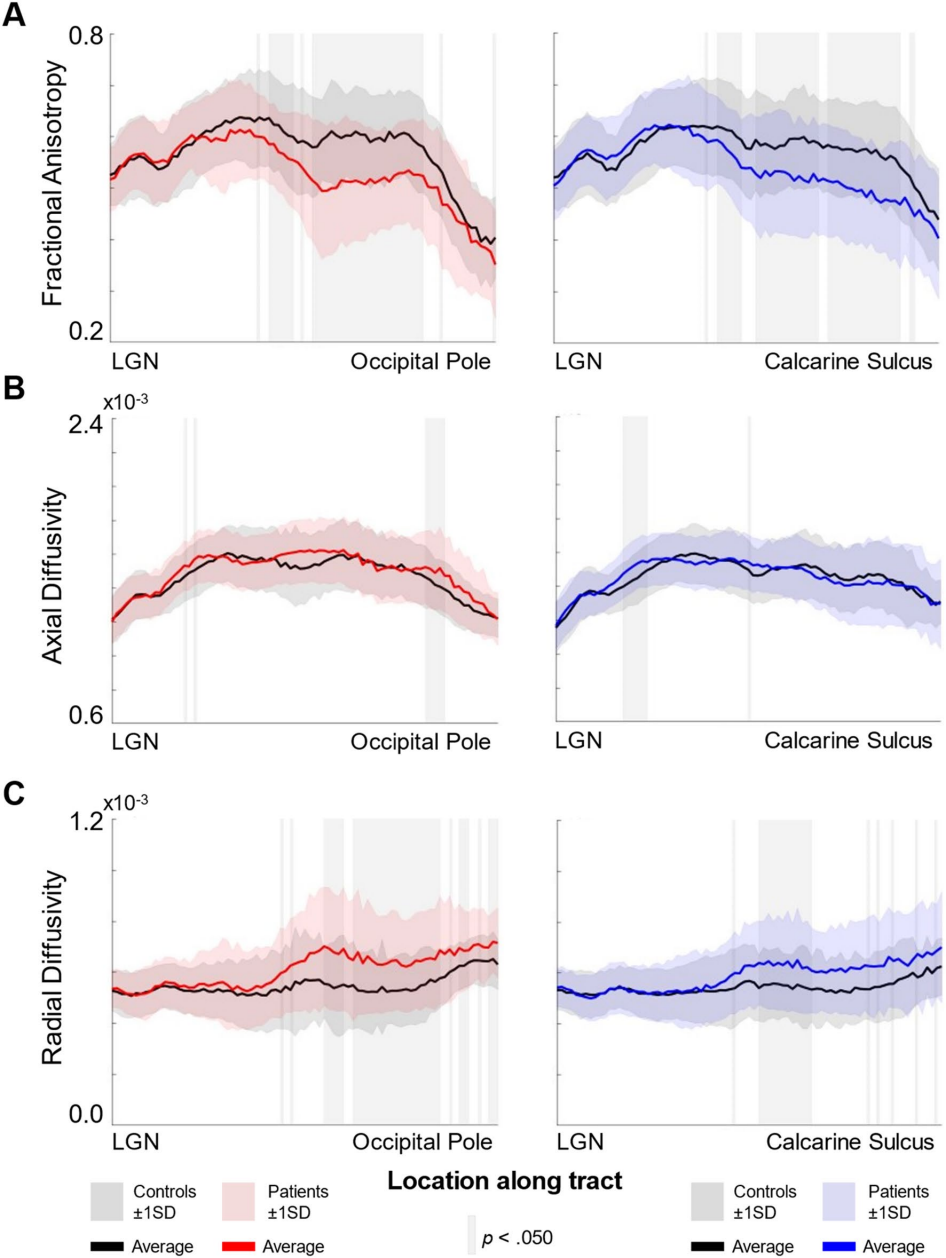
(**A**) Mean fractional anisotropy (FA) for controls (black line) and MD patients (red/blue lines). (**B**) Mean axial diffusivity (AD) measures for controls and MD patients. (**C**) Mean radial diffusivity (RD) measures for controls and MD patients. Standard deviation is shown in the shaded regions surrounding the group mean lines (dark grey for controls, red/blue for MD patients). Data are presented for our projections to: (from left to right) the occipital pole and calcarine sulcus. Locations along the tract profile that emerged significantly different between groups is shown in shaded grey columns (*p* < 0.050).

We wanted to determine if there were group differences at certain locations along the tracts for fiber bundles projecting to ROIs in visual cortex, and whether these would be greater in tracts projecting to the occipital pole compared to the calcarine sulcus. After averaging across time points, we then averaged across participants to generate group tract profiles for MD patients and sighted controls (Fig. 5A). A mixed ANOVA revealed a significant interaction between ROI (occipital pole vs calcarine sulcus), group (MD vs controls) and location along the tract (*F*(80,2160) = 1.371, *p* = 0.018) for our mean FA data. To tease apart these main effects, we ran two one-way ANOVAs (one for each ROI) correcting for multiple comparisons, reporting Welch’s F values to accommodate unequal variances. For the tract projecting to the occipital pole, we found significant differences in 34 locations along the tract (all in range *p* ≤ 0.001 to *p* = 0.039). For the tract projecting to the calcarine sulcus, we observed significant differences in 39 locations (all *p* ≤ 0.001 to *p* = 0.049). All significant locations are shown in Fig. 5A, indicated by the light grey blocks. Data suggest that there is a consistent drop in FA in a portion of the tracts closer to early visual cortex.

Having identified the locations of the tract where FA was significantly lower in MD patients compared to sighted controls, we next wanted to determine whether we see a change in FA over time in this portion of the tract. To assess possible changes, we entered data for each individual time point into a linear mixed effects model in the same manner as described for cortical thickness and myelin density measures.

As shown in Table 2, age at baseline was a significant predictor of FA for the projections to both ROIs (occipital pole: *t*(24.99) = − 6.55, *p* < 0.001, *d* = − 2.62; calcarine sulcus: *t*(25.13) = − 6.24, *p* < 0.001, *d* = − 2.49), however despite this, group also emerged as a significant predictor for both projections (occipital pole: *t*(26.15) = − 2.21, *p* = 0.036, *d* = − 0.86; calcarine sulcus: *t*(26.38) = − 2.11, *p* = 0.044, *d* = − 0.82). There was no significant effect of time (occipital pole: *t*(49.12) = − 1.26, *p* = 0.214, *d* = − 0.36; calcarine sulcus: *t*(49.26) = − 1.04, *p* = 0.302, *d* = − 0.30), nor were there any group × time interactions for either projection (occipital pole: *t*(49.43) = − 1.37, *p* = 0.178, *d* = -0.39); calcarine sulcus: *t*(49.59) = − 0.54, *p* = 0.590, *d* = − 0.15), indicating that during the time window of the study there was not a significant change in FA in either group, although the reduction in FA over time was greater in the patient group.

#### Axial and radial diffusivity

Whilst FA gives us a sense of the structural integrity of the tracts of interest, it cannot tell us about the nature of the changes observed. It is therefore important to also examine AD and RD to determine what may be driving the FA decrease observed in our patient group. To allow us to compare against FA measures, we ran two one-way ANOVAs (one for each ROI), correcting for multiple comparisons and reporting Welch’s F values. For AD, it appears there are few differences between sighted controls and MD patients. The number of locations that emerged significantly different between groups were as follows: 7 for the occipital pole projection (in range *p* ≤ 0.004 to *p* = 0.044), and 2 locations for the calcarine sulcus (in range *p* = 0.038 to *p* = 0.045). All significant locations are illustrated by grey shaded blocks in Fig. 5B.

We observed significant differences in RD between groups in locations that largely overlapped with those that were significant when assessing FA; this is true for projections to the occipital pole (34 locations, all in range *p* = 0.003 to *p* = 0.042) and calcarine sulcus (28 locations, all in range *p* = 0.010 to *p* = 0.048). Shaded blocks in Fig. 5C indicate the locations where MD patients showed significantly greater RD measures compared to sighted controls. For a detailed report of the inferential statistics for FA, AD and RD for every location along our tracts of interest, see Tables 1–1 in Supplementary Information.

## Discussion

Our study aimed to assess the status of the posterior visual pathway in bilateral macular degeneration (MD), by acquiring multiple anatomical measures on both MD patients and controls over a ~ 2-year period. Supporting the ageing literature, all participants irrespective of group did show a decline over time in cortical thickness and fractional anisotropy (FA). However, we also observed significant group effects whereby patients show a reduction in cortical thickness for both ROIs, a reduction in FA in the optic radiations, and a significant increase in rate of loss of cortical myelin density for the patient group also emerged. Overall, we see a consistent and predicted change in a negative direction across our anatomical measures, and moderate effect sizes for group by time interactions for both the cortical thickness and FA data hint that patients may also be showing a faster decline for these measures, however these did not reach significance.

### Changes in cortical thickness

These results support and extend previous cross-sectional studies, which have highlighted reductions associated with natural ageing, and also largely supports previous work reporting a thinning of cortex in the posterior visual pathway in MD^1,3,5,8,10,12,16,17^. The group data for cortical thickness support earlier work in our lab; Hanson et al.^17^ followed a group of unilateral AMD patients and found a reduction in volume of the occipital lobe, driven by cortical thinning in the occipital pole. Furthermore, in a separate cohort of bilateral wet-AMD patients, Hanson et al.^16^ found the occipital pole and calcarine sulcus both showed significant thinning. Here, we report cortical thinning in the same ROIs in a different cohort of bilateral MD patients. As stated previously, without quantitative assessments of patient scotoma size and retinotopically defined ROIs in visual cortex, we cannot rule out the possibility that more subtle differences may be observed in representations of more peripheral visual field locations as well. The effect size for the group difference was smaller for this representation (− 0.79) than for the occipital pole (− 1.34) suggesting a greater group effect in the occipital pole. We cannot ignore the fact that, even though it is not significant, data suggest patients with established bilateral MD are still showing a faster decline in visual cortex, moderate effect sizes for both the occipital pole and calcarine sulcus (0.44 and 0.46 respectively) indicate that a larger scale longitudinal study may reveal this.

While not significant, results hint that in the calcarine sulcus, the thinning of cortex seems to be faster in the control group compared to the MD patients. Burge et al.^15^ report that, in patients with central field loss, the representation of the intact peripheral visual fields was thicker, this was attributed to the increased reliance of the remaining peripheral vision, arguing it may be indicative of visual cortex compensating for the loss of central vision. While our cross-sectional work and the group difference we report here is not consistent with this finding, it may be possible that over time, a faster thinning in controls could result in a group difference where patients’ cortex is thicker than in controls.

### Changes in cortical myelin density

**Figure 6:**
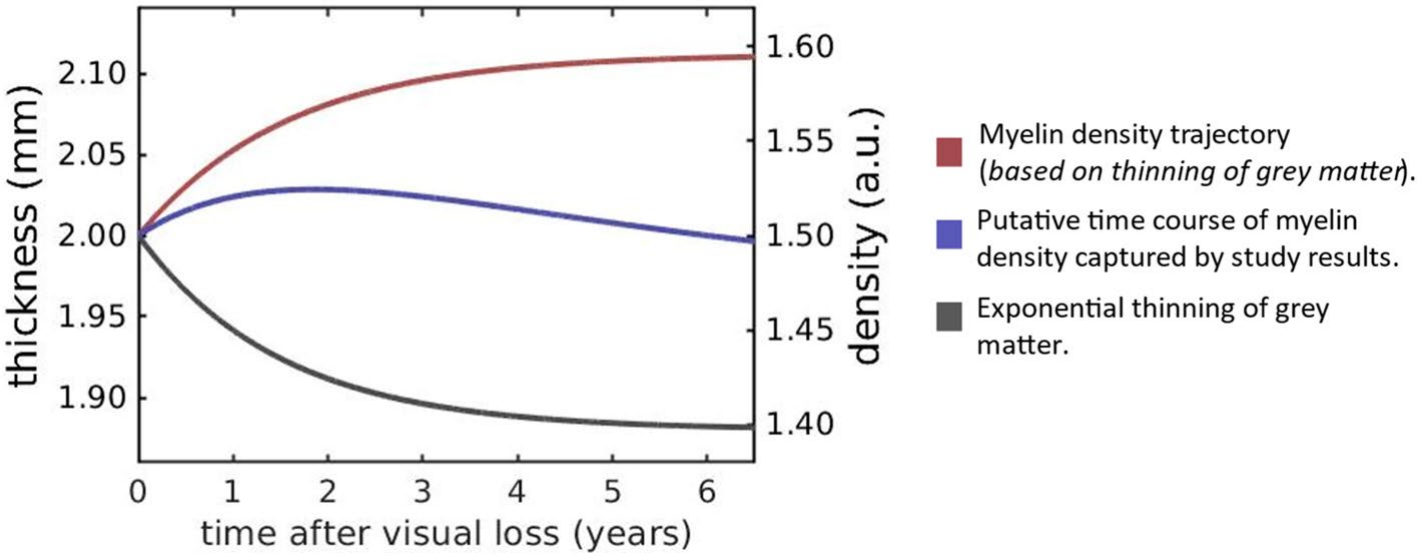
Time course of cortical atrophy. The grey line shows an exponential thinning of grey matter as measured on the left-hand axis. The red line shows how a constant volume of myelin would register as an increase in myelin density (right hand axis) because of the thinning of grey cortex (shown by the grey line). The blue line shows a putative time course of myelin density that first increases because the thinning of cortex dominates, but later decreases because of a genuine loss of myelinated tissue. The blue line therefore captures our results qualitatively; the patient group has a larger myelin density compared to controls, but the density is falling faster over time.

At face value our finding that myelin density is higher in patients than controls seems counterintuitive. It is likely, however, that the proportion of cortical myelin relative to grey matter may be high in MD patients due to the observed reduction in grey matter rather than MD patients showing an absolute increase in cortical myelin for which there would be no apparent explanation. It appears a similar age-dependent atrophy of cortex is accompanied by a relative increase in myelin in the longitudinal myelin density control data (as shown in rate of change data in Fig. 4B–D); this is a pattern observed in previous work also^36,52–55^. In addition to the higher myelin density in patients compared to controls, we also found that time affected myelin density of the participant groups differently; relative to controls myelin density reduced over time in patients. We interpret this on the basis that cortical thickness and cortical myelin atrophy at different rates over time. If for example cortical thickness reduces more quickly, it will inflate myelin density, but those inflated values will fall over time because of a pathological atrophy of myelin, which we propose occurs more slowly. A model of the two processes is shown in Fig. 6, where we show how, if myelinated cortical tissue is unaffected, myelin density will be inflated in a way inversely proportional to the thickness of cortex. This is consistent with previous studies and the group differences we report. However, it is not consistent a loss of myelin density over time in the patient group relative to controls. We therefore show that if in addition to accounting for myelin density changes resulting from cortical thinning, we add an atrophic, but slower, change to myelinated tissue, both an initial increase in myelin density followed by a slow reduction of it over time would be observed.

In addition to the interplay between measures described, a thinner cortex and reduction in FA overall in patients across ROIs could have been explained by the age gap between our participant groups. However, we accounted for this by including age at the time of enrolment into our model. Despite revealing that age was a significant predictor for both measures, our group and time effects still emerged and therefore age cannot solely explain the thinner cortex in MD patients.

### When does cortex thin?

The MD patients included in the current study are a heterogeneous group with varying diagnoses, however what they have in common is a bilateral loss of central vision. The ~ 2-year time window we are exploring here is for most, a considerable time after diagnosis and after sight loss. This is an important window to assess, as it helps to characterise disease progression in the later stages, determining whether changes still occur or whether cortex stabilises. However, it is also important to consider the earliest stages of the disease as well. Hanson et al.^17^ showed in a separate cohort of unilateral wet AMD patients, that no significant change emerged for cortical thickness approximately 3–4 months after diagnosis of AMD in the fellow eye, but significant reductions in thickness in the occipital pole, and overall volume of the occipital lobe were observed after approximately 5 years. To fully understand the nature of the changes that occur within those first 5 years, multiple measurements on individuals in the first few years post diagnosis will help us to better characterise the time course of changes in visual cortex. As is evident in the current study, matching patients by age, disease type and onset is a highly demanding task, but characterising changes over time in individuals with varying diagnoses and disease severity can still be fruitful. We had a unique opportunity to assess cortical thickness immediately before, and the first 2 years after diagnosis in an individual—MD7—as this individual had participated in research at the centre prior to an AMD diagnosis. MD7 lost a substantial amount of vision due to bilateral AMD and a secondary bleed in one eye. As shown in Supplementary Fig. 1, data suggest a decline over the time points immediately after diagnosis (Supplementary Fig. 1A), and the rate of change for this participant was not captured by the group data, as values exceeded that of both control and MD patient groups (Supplementary Fig. 1B). This gives us an indication that perhaps greater cortical thinning occurs at the early stage of the disease (although likely not as soon as 4 months—Hanson et al.^17^) after sight loss, but stabilises over time.

### Changes in white matter tracts—the optic radiation

The reductions in FA observed extend findings reported by Yoshimine et al.^22^ whereby MD patients showed a significant reduction in FA in the optic radiations in a portion of the tract projecting to the ROI capturing the central representation—the occipital pole for this study. We also show that this is likely driven by an increase in radial diffusivity (RD), which emerged significantly higher in MD patients in similar locations along the tract. However, we also observed this pattern in the projection to the more peripheral visual field representations—the calcarine sulcus. This may be the due to the fact different atlases were used, Yoshimine and colleagues used the Benson atlas^56,57^, and divided V1 into 3 portions capturing the fovea, mid-peripheral and far periphery. Our ROIs were taken from FreeSurfer’s Destrieux atlas which are not restricted to V1, but we also collapsed across a larger portion of the peripheral field representations. We opted for the Destrieux atlas ROIs to keep consistent with those used to explore cortical thickness and myelin density changes, but also to keep consistent with other work published in the lab^16,17^. When looking at individual tract profiles in Yoshimine et al.^22^, it does appear that AMD patients on average exhibited lower FA in the peripheral representations in a portion of the tract, however it did not reach significance. Our patient group, while larger, also consists of individuals with bilateral vision loss. The optic radiation fibres that project from the LGN to visual cortex are monocular, and therefore the severity of vision loss may affect the extent of changes observed in the posterior visual pathway and as described previously, we did not have visual field assessments for our patients, nor were our ROIs retinotopically defined. In support of this, Malania et al.^8^ noted that it was not just the central projections that were showing a reduction in FA in patients with central loss in their cohort, suggesting more widespread changes to the visual white matter, as we have shown here.

The reduction in FA and corresponding increase in RD coincides with findings from previous work on partial vision loss, for both central and peripheral affecting pathologies^22,58–60^. As with cortical thickness and myelin density, it is hard to determine what the atrophy observed reflects, and for FA, we also have to make inferences based on the diffusion measures reported. Early mouse models report that impaired white matter can be a result of demyelination, axonal injury or a combination of the two^61–64^. Demyelination was associated with increases in RD, whereas axonal injury was associated with reductions in AD. The inverse can be seen in human development, whereby an increase in FA was coupled with a decrease in RD^21,23,52^.

### Limitations and future directions

While the current study has provided insights into the atrophic changes occurring in the posterior visual pathway, there are some limitations. For our control cohort, some individuals were scanned on a different scanner and some using different protocols but on the same scanner as the MD patients. Data from the Siemens scanner were only incorporated in analysis to explore the relationship between cortical thickness and myelin density, and converted to Z scores within scanner to accommodate for this. Additionally, the subset of controls (C13–C18) who were scanned on the GE scanner (same as MD patients) with a different protocol were originally enrolled in another study. For this, we replicated the scanning protocol from the original study to ensure consistency within individuals. Furthermore, the limitations of the funding period meant the window in which we could collect data was narrow. As well as typical attrition, the staggered recruitment of our MD patients also led to patients completing a different number of scanning sessions. Linear mixed effects modelling allowed us to include each individual data point and the time since the first scan as a fixed effect, we were more constrained to using linear models given we only had two time points for controls. The ideal scenario would therefore be to scan controls at the same number of time points, which would also allow us to consider non-linear models when exploring changes over time.

### Concluding comments

As the human population is ageing, and most ophthalmic pathologies develop late in life, we need to understand the nature of changes in the entire visual pathway, from eye to brain, but particularly in AMD given it is currently the leading cause of blindness in the developed world^65,66^. As highlighted by Hanson et al.^16^, determining which features of eye disease drive atrophy in the posterior visual pathway is important, as plasticity will be brought into question—is the mature brain capable of responding appropriately if visual function were restored? We also need to determine whether it is sufficient to treat the eye alone, or whether we need to incorporate additional neuroprotective strategies to prevent or slow the cortical changes. Evidence already indicates the latter is necessary^5,12,14–17,67^. Further longitudinal assessments in age-matched sighted individuals will provide the necessary context for understanding how the posterior visual pathway responds to ageing naturally and build a model of how AMD might progress.

We conclude that there is accumulating evidence of the thinning of visual cortex in individuals with MD, which continues throughout the disease progression, as shown in cases of long-standing bilateral macular degeneration in this study. Cortical myelin is also showing possible signs of decline in early visual cortex, more so in the representation of the visual field defect, something which has not been demonstrated before to our knowledge. White matter projections to early visual cortex also showed signs of reduced integrity. Changes were broad, affecting bundles projection to both central and peripheral representations. Establishing the link between changes in the anterior and posterior visual pathways is important, as while it seems likely that structural and functional changes in the retina drive observed changes in the brain, the causes of these structural changes are not fully understood. Therefore, monitoring the entire visual pathway in individuals in the earliest stages of the disease will provide valuable insight into the time course of changes, and help determine at what point during disease progression interventions would be most effective at slowing atrophy.

## Supplementary Information

Supplementary Information.

## Data Availability

Participants consented to the use of their data by the investigators and to third parties, but only after the use of the data by those third parties was scrutinised by the York Diagnostic Imaging Centre's Research Governance Committee. The corresponding author may be contacted to initiate data sharing and assistance with seeking ethical approval for the use of the data.
