# Supplementary material for: Assessing the structure of the posterior visual pathway in bilateral macular degeneration
Source: Sci Rep. 2023 Mar 27;13:5008. doi: 10.1038/s41598-023-31819-x (PMC10042846; doi:10.1038/s41598-023-31819-x)
Supplement: Supplementary file 1 — Supplementary Information. [file 41598_2023_31819_MOESM1_ESM.docx]

**Supplementary Information.**

**Assessing the structure of the posterior visual pathway in bilateral macular disease**

Holly D. H. Brown^a,b,c,f^, Richard P. Gale^d,e^ , André D. Gouws^b^, Richard J. W. Vernon^a,b,c^, Archana Airody ^e^, Rachel L. W. Hanson^a,b,c,e^ ,Heidi A. Baseler^a,b,c,d^, Antony B. Morland^a,b,c^

**Affiliations**:

1. Department of Psychology, University of York, York, UK
2. York Neuroimaging Centre, University of York, York, UK
3. York Biomedical Research Institute, University of York, York, UK
4. Hull York Medical School, University of York, York, UK
5. Academic Unit of Ophthalmology, York and Scarborough Teaching Hospital NHS Foundation Trust, York, UK
6. Centre for Cognition and Neuroscience, Department of Psychology, University of Huddersfield, Huddersfield, UK

**Corresponding Author:**

Dr Holly D. H. Brown

h.d.brown@hud.ac.uk

Department of Psychology, School of Human and Health Sciences, University of Huddersfield, HD1 3DH

**
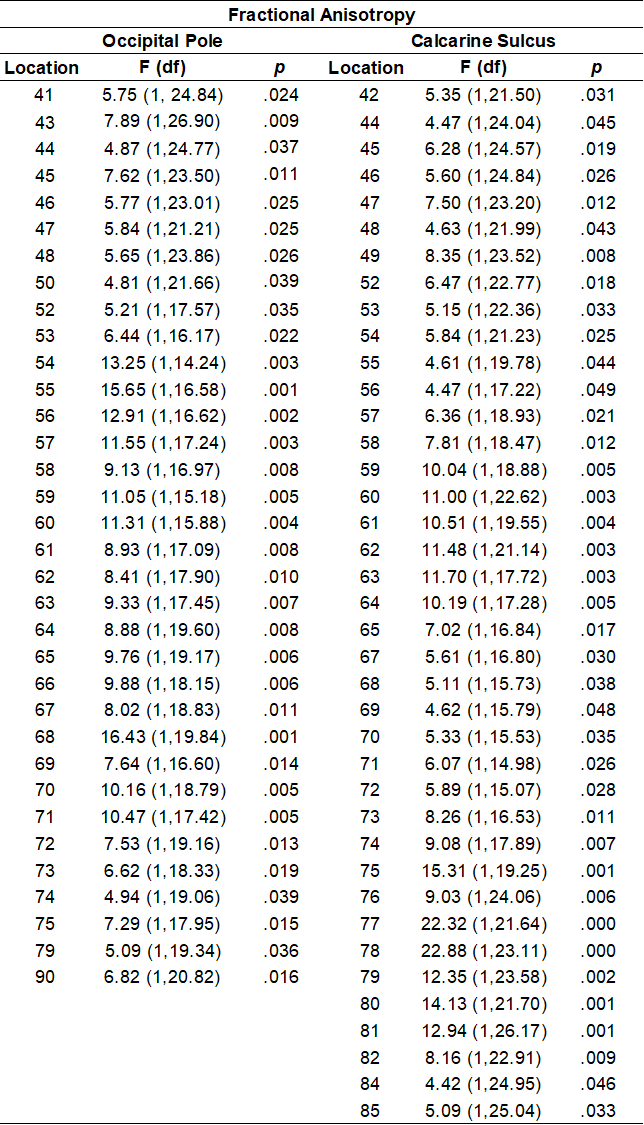
Supplementary Table 1:** Full output for one-way ANOVA for fractional anisotropy for each ROI (occipital pole and calcarine sulcus), correcting for multiple comparisons and reporting Welch’s F values.


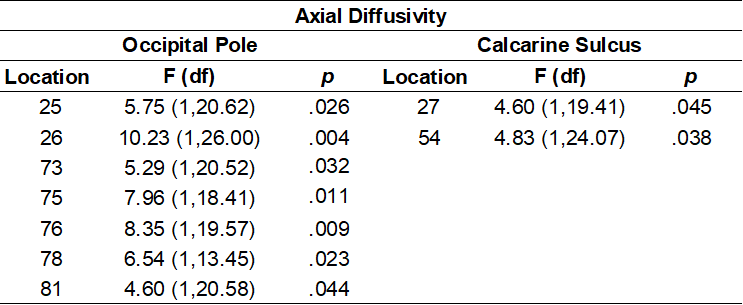
**Supplementary Table 2:** Full output for one-way ANOVA for axial diffusivity for each ROI (occipital pole and calcarine sulcus), correcting for multiple comparisons and reporting Welch’s F values.


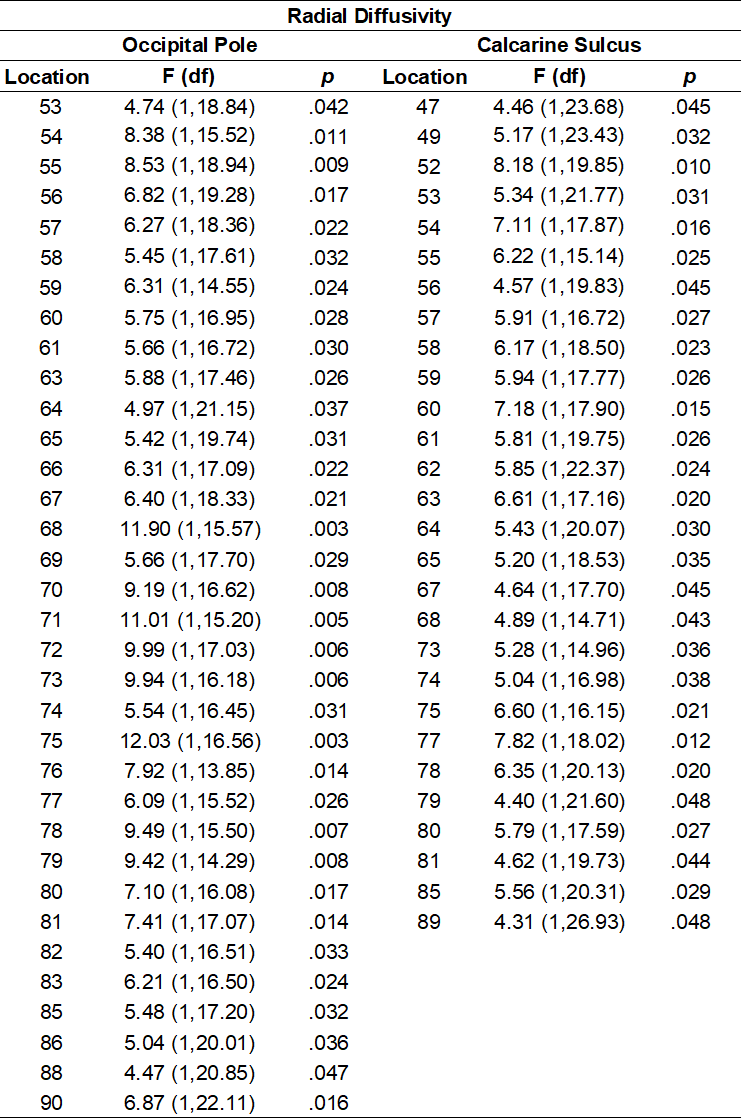
**Supplementary Table 3:** Full output for one-way ANOVA for radial diffusivity for each ROI (occipital pole and calcarine sulcus), correcting for multiple comparisons and reporting Welch’s F values.


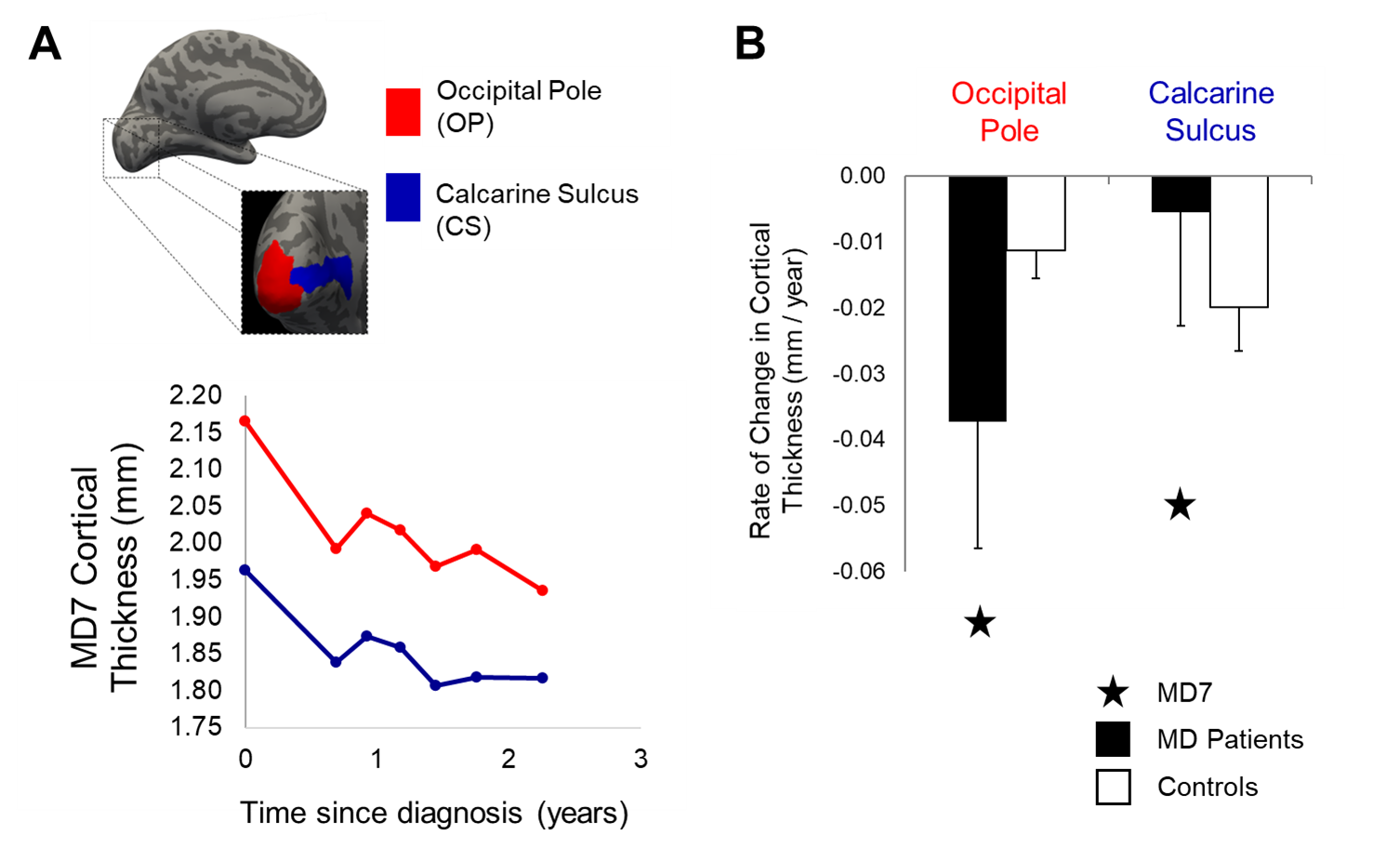
**Supplementary Figure 1:**

**A:** Upper part: ROIs occipital pole (red) and calcarine sulcus (CS) displayed on the inflated cortical surface. Lower: Mean cortical thickness (mm) for MD7 at each time point since diagnosis (time zero). **B**: MD7 rate of change in cortical thickness data (stars) displayed in the context of the MD and control group data. Here, we can see a much greater negative rate of change for both ROIs for MD7, not captured by the MD group data (black bars).
